# Hooked on you: shape of attachment structures in cymothoid isopods reflects parasitic strategy

**DOI:** 10.1186/s12862-019-1533-x

**Published:** 2019-11-08

**Authors:** Charles Baillie, Rachel L. Welicky, Kerry A. Hadfield, Nico J. Smit, Stefano Mariani, Robin M. D. Beck

**Affiliations:** 1 School of Biological and Environmental Sciences, Liverpool John Moores University, Liverpool, L3 3AF UK; 2 School of Aquatic and Fishery Sciences, University of Washington, 1122 NE Boat St, Seattle, 98105 USA; 3 Water Research Group, Unit for Environmental Sciences and Management, North-West University, Potchefstroom, 2531 South Africa

**Keywords:** Attachment, Convergence, Isopod, Parasite, Tongue-biters

## Abstract

**Background:**

Parasite attachment structures are critical traits that influence effective host exploitation and survival. Morphology of attachment structures can reinforce host specificity and niche specialisation, or even enable host switching. Therefore, it is important to understand the determinants of variation in attachment structures. Cymothoid isopods are striking ectoparasites of fishes that include the infamous ‘tongue-biters.’ They are known to parasitise hosts in one of four qualitatively distinct anatomical regions. Here, we quantify variation in cymothoid attachment structures — hook-like appendages called dactyli — and test whether differences in dactylus shape are correlated with parasite mode (where they attach), allometry, or both, using multivariate ordinary least squares regression. We also assess the influence of shared ancestry on shape using a molecular phylogeny to weight our models using phylogenetic generalised least squares regression.

**Results:**

We find clear differences in shape between externally-attaching and internally-attaching cymothoids but also between anterior and posterior dactyli across various species with the same attachment mode. Allometric effects are significant for anterior but not posterior dactyli. Mouth-attaching species show greater shape variability than gill- and mouth-attaching species. We find no evidence that there are clade-specific patterns of association between parasite mode and dactylus shape.

**Conclusions:**

Parasite mode appears to be the main driver of attachment morphology. This likely reflects several components of parasite ecology including feeding and functional demands of attachment in different microhabitats. Geometric morphometric approaches to the quantification of shape variation of simple structures is an effective tool that provides new insights into the evolvability of parasite attachment.

## Background

Permanent ectoparasites derive almost all of their energy and habitat requirements from a single host source [1]. Thus, traits for attachment function, which are imposed by this lifestyle, are critical for parasite survival and reproduction. These traits are often ecomorphologically significant, segregating species between different host niches [2, 3]. Morphologies shared by parasite species are likely, then, to reflect similarities in their host use. In a co-evolutionary context, attachment traits may also drive specialisation of location upon a host, partly define the limits of a parasite’s host range (i.e. the breadth of host species it could infest), or enable permanent switches to different host species entirely [4].

A remarkable example of permanent ectoparasitism is seen in the family Cymothoidae, of which all known species are obligate parasites of fishes [5]. Individuals of the infamous *Cymothoa exigua* supplant their hosts’ tongues — the only example in nature of ‘anatomical replacement’ by another organism [6]. Four attachment modes can be recognised within Cymothoidae: (1) mouth-attaching species (including the ‘tongue-biters’), (2) gill-attachers found in the branchial chamber, (3) skin-attachers, those attached externally to the scales or skin, and (4) flesh-burrowing species that encapsulate themselves within their host’s body cavities [7]. Whereas gill-, mouth- and flesh-attachers are predominantly marine species, flesh-burrowing cymothoids are typically freshwater species.

Parasitic strategy is largely conserved within cymothoid genera [5, 8], but there is substantial variation in microhabitat use between species with the same parasitic mode, because distinct locations or orientations are used by different species. For example, at the genus level, *Anilocra* and *Nerocila* comprise exclusively skin-attaching species, but are found on the anterior and posterior regions of their hosts, respectively [9, 10]. Within *Anilocra*, species are often site specific across the anterior region, for example *A. haemuli* is always found attached near the eye, while *A. acanthuri* attaches under the mouth [10]. Very few species are known to use more than one attachment mode, with all such examples recorded from atypical host associations. For example, the most common Brazilian freshwater species, *Braga patagonica*, is a branchial parasite of several fish species but on cultured *Colossoma macroponum* it is regularly recorded externally attached behind the dorsal fin [11]. The evolution of parasitic mode within Cymothoidae remains unclear, but there is some consensus that each mode has evolved more than once, and that skin-attachment is unlikely to be ancestral for the group as a whole [12, 13].

Cymothoids are well adapted for ectoparasitism on their mobile fish hosts; a thickened cuticle affords protection; increased surface area of gill-bearing pleopods facilitates oxygen transfer in gill- and mouth-attaching species, and modified mouthparts enable the acquisition of blood meals in skin-attachers [7, 14]. Externally-attaching species, relative to other parasitic modes, exhibit dorso-ventral flattening, which reduces drag and minimises the energy expenditure of their hosts [14]. Crypsis is also displayed by some externally-attached species as a strategy to avoid predators such as cleaner fish [15]. The appendages cymothoids use for attachment are particularly characteristic: each of their prehensile pereopods, ‘walking’ limbs, terminate in a recurved dactylus, presence of which is a synapomorphy of cymothoid species [8]. Cymothoids and their close relatives exhibit a wide spectrum of trophic dependency from free living species, through temporary to obligate parasites, and cymothoids are thought to have evolved from either a cirolanid-like or an aegid-like ancestor [7, 8, 16]. Species in the families Cirolanidae and Aegidae do not possess recurved dactyli on their posterior pereopods, and adults retain their ability to swim [17]. In contrast, cymothoids lose the ability to swim after they have infested a suitable host, which drastically reduces the probability of finding another host in the event of being displaced [1]. Loss of swimming appendages may have evolved in concert with the origination of cymothoid dactylus morphology as a trade-off between an increasing reliance on host resources and maintenance of traits for the acquisition of new host individuals [14].

As an important trait for facilitating obligate ectoparasitism in cymothoids, we hypothesised that variation in dactylus shape would reflect differences in the functional demands of parasitising hosts in different locations. Gill- and mouth-attaching cymothoid species use their dactyli to penetrate host tissue, but also as hooks to clasp gill-rakers, tongues, or the upper palate [5, 18–20]. In contrast, externally-attaching cymothoids use dactyli to anchor themselves to host musculature and dermal tissues and are subject to greater hydrodynamic forces. We predicted that the externally-attaching species would have dactyli that are relatively longer, thinner, and ’needle-like’ adapted for piercing flesh, while those of gill- and mouth-attaching species will be stouter, more recurved, and strengthened for ’gripping’. To test these predictions we used a geometric morphometric approach to quantify dactylus shape and assessed the influence of parasitic mode, size allometry, and phylogeny on shape variation.

## Methods

### Specimens

Cymothoid specimens used in this study are from collections at the Water Research Group, North-West University, Potchefstroom, South Africa, and the University of Salford, Manchester, UK. We took images of the first pereopod (*P*1) from 124 individuals across 18 species, and from 135 individuals of 19 species for the seventh pereopod (*P*7). Only *P*1 and *P*7 were measured, since these are recognised as the most useful for taxonomic studies because of considerable morphological variation between species; *P*2−6 show much less shape variation between species [20–22]. All specimens were adult females, each species was represented by at least three individuals, and there was a minimum of 26 individuals for each of three parasitic modes (Table 1). Flesh-burrowing specimens were not included due to insufficient sample numbers and because the seventh pereopods do not possess a recurved dactylus, only a simple stub [23]. All sampled species are known to occur in the ocean around southern Africa except for *Anilocra chromis* and *Anilocra physodes* which are found in the Caribbean and Mediterranean, respectively.

**Table 1:** Parasite specimens landmarked for this study

Parasitic mode	No. of individuals		No. of species	
	*P*1	*P*7	*P*1	*P*7
External	26	38	5	6
Gill	37	36	6	6
Mouth	61	61	7	7
Total	124	135	18	19
Species	*P*1	*P*7	Mode	GB Acc.
*Anilocra capensis*	11	11	E	**MK652475**
*Anilocra chromis*	3	4	E	KY562736
*Anilocra physodes*	3	3	E	**MK652476**
*Ceratothoa africanae*	10	9	M	**MK652477**
*Ceratothoa carinata*	6	6	M	**MK652479**
*Ceratothoa famosa*	10	9	M	Not Available
*Ceratothoa retusa*	7	10	M	**MK652478**
*Cinusa tetrodontis*	10	10	M	**MK652480**
*Cymothoa eremita*	8	8	M	**MK652481**
*Cymothoa sodwana*	10	9	M	**MK652482**
*Elthusa raynaudii*	9	7	G	**MK652487**
*Elthusa sp.*	3	4	G	Not Available
*Mothocya affinis*	9	8	G	**MK652484**
*Mothocya plagulophora*	3	3	G	**MK652483**
*Mothocya renardi*	7	7	G	**MK652485**
*Nerocila depressa*	4	5	E	MH425627
*Nerocila sigani*	0	10	E	Not Available
Gen. nov. et sp. nov.	5	5	E	Not Available
*Norileca indica*	6	7	G	MF628259

E = External, G = Gill, M = Mouth. GB Acc. a re Genbank accession numbers for molecular sequences. Newly generated sequences are in bold typeface

### Image and landmark acquisitions

**Fig. 1:**
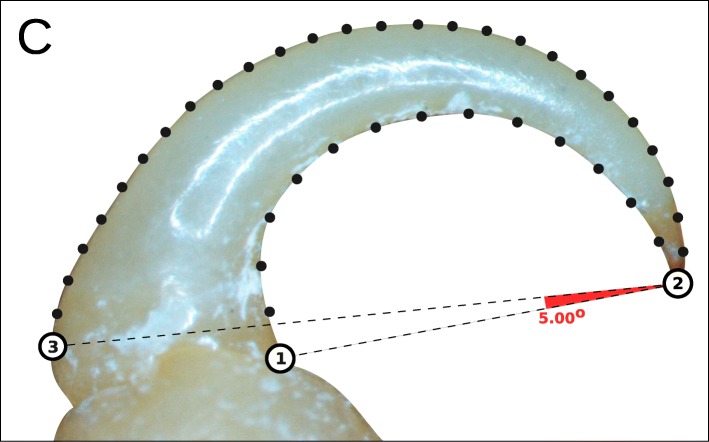
Landmark design for cymothoid dactyli. Numbered circles represent location of full landmarks and black points are semi-landmarks. Dashed lines measure a 5^*o*^ angle between landmarks 1 and 2, from which landmark three was positioned

We captured high resolution digital images of the *P*1 and *P*7 dactyli for each individual using a Nikon DS-Fi1 camera fitted to a Nikon SMZ1500 stereoscopic microscope. For each of *P*1 and *P*7, we plotted 39 semi-landmarks to describe two curves between three fixed landmarks (Fig. 1) with tpsDig2 [24]. The first fixed landmark was located at the medial junction with the propodus, and the second landmark was placed at the distal tip of the dactylus. Due to differences between individuals in how the propodus overlaps and obscures the dactylus, we drew a line between these first two landmarks, and another at a 5 ^*o*^ angle from this. The third fixed landmark was placed on the lateral edge of the dactylus at the intersection of the 5 ^*o*^ line, thus removing joint shape information from the same relative point in each specimen. The first curve was plotted between the first and second landmarks, along the medial edge of the dactylus, re-scaled by length with 13 semi-landmarks. We used the same method for the second curve, between the second and third landmarks, but with 26 semi-landmarks as this edge is between 150-200% the length of the medial curve. Thirty images were plotted a second time to calculate landmarking error.

### Phylogeny reconstruction

We generated a new phylogenetic tree using molecular sequence data from the barcode region of mitochondrial Cytochrome Oxidase subunit I (COI: [25]). A sequence was obtained from one representative of each species, for which an image was captured, except *Ceratothoa famosa*, *Elthusa sp.*, *Nerocila sigani*, and gen. nov. et sp. nov. There are no publicly available sequences for these species, and the preservation condition of our specimens did not produce DNA sufficient for PCR. For better preserved specimens, we extracted DNA from a single pereopod using a Machery-Nagel spin column kit, before amplification with Polymerase Chain Reaction (PCR) following the protocol in [22]. Each PCR product was purified, then sequenced in both directions on an ABI 3630 Genetic Analyzer, and we generated consensus sequences with Geneious R10 (https://www.geneious.com). We added sequences for *Nerocila depressa* (MH425627) and *Anilocra chromis* (KY562736), and used the aegiid, *Aega psora* (FJ581463), as an outgroup. We aligned nucleotide sequences with TranslatorX [26] and used Gblocks v0.91b [27] to remove ambiguously aligned sites resulting in a trimmed alignment of 581bp.

Due to the length of alignments and our concern that phylogenetic signal might be limited, we applied a backbone constraint to our tree searches based on the latest available molecular phylogeny for cymothoids [12]. This allowed us to better place taxa for which we have no phylogenetic information and to calculate branch lengths. Specifically, these included hard constraints on genera represented by more than two species, while restricting the potential placement of other taxa within those clades. The best fitting substitution model for the alignment (GTR + *Γ*) was selected using corrected Akaike’s Information Criterion (AICc) in jModelTest 2.1.10 [28, 29]. Tree searches were performed under a Maximum Likelihood (ML) framework with RAxML v8 [30], and topological support was assessed with 500 rapid bootstrap replicates. The topology of the constrained tree was then compared against the unconstrained topology using the Shimodaira-Hasegawa test [31]. We ultrametricised the tree using the function ’chronos’ of the ape package in R under a correlated trait model, and an optimised value for the smoothing parameter (from 5,000 starting values) was selected as that which produced the tree with the highest penalised log-likelihood [32].

### Statistical analyses

All subsequent analyses used R v3.4.4 [33] and the packages ape 5.2 [34], geomorph 3.0.7 [35], phytools 0.6 [36], nlme 3.1 [37] and stats [33]. We obtained individual shape variables from our raw landmark coordinates by Generalised Procrustes Analysis (GPA; [38], using Procrustes distance to optimise locations of semi-landmarks [39]. This superimposition produces a set of scaled and aligned Procrustes coordinates that minimises location, orientation, and size differences between samples, thus retaining only information related to geometric shape. The mean consensus shapes for each species in both the *P*1 and *P*7 configurations were aligned in a second GPA to produce species level Procrustes coordinates [40]. We then performed Principal Components Analysis (PCA) on the aligned coordinates to visualise shape differences plotted as morphospaces and PCA backtransformations [40]. The PCA scores were also used as shape variables in regression analyses, for which we retained the first twelve principal components with non-zero eigenvalues. Together these explained over 99% of variation in both our *P*1 and *P*7 datasets. For phylogenetic analyses we generated sets of shape variables for *P*1 and *P*7 with the same method including only the species present on our phylogeny, and using phylogenetic PCA (pPCA) - *P*1_*phy*_ and *P*7_*phy*_ datasets.

At this point, we quality-checked our full *P*1 and *P*7 datasets to identify landmark or analytical problems. First, for each dataset, we calculated the mean Procrustes distance of each sample to the *P*1 or *P*7 global consensus shape, where outlying data points might indicate landmark error. Outliers were retained if they were consistent within a species. For example, in the *P*1 dataset individuals of *Norileca indica* all appear above the upper quantile, which reflects genuine shape information, rather than error. Error in our data acquisition steps was assessed by nested ANOVA between our 30 repeated landmark sets to calculate the ratio between total Mean Squared Error (MSE) and that contributed by our replicates. We found our digitisation to be over 97% repeatable, and, therefore, that measurement error did not significantly influence variation in shape.

For both our *P*1 and *P*7 datasets, we first fitted a ’full’ model including all covariates and their interactions, with single factor non-parametric Multivariate Analysis of Covariance (Procrustes npMANCOVA). The twelve principal components describing shape formed our response and as predictors we modelled size as a continuous covariate, parasitic mode as a three-level factor, and the interaction between size and mode. For size, we used the mean of log-transformed centroid sizes for each species, which are the square root of the sum of squared distances between the landmarks of each specimen and their centroid [41]. We then proceeded with pairwise tests to assess homogeneity of slopes (whether patterns of shape-allometry are common across parasitic modes), and group means (testing shape differences between groups after accounting for variation in size). Finally, for both datasets we also used the residuals from a regression of shape on size (equivalent to allometry-free shapes) to test shape differences between parasitic modes. This latter analysis is only appropriate where there is a common allometry between groups [42]. For each analysis we used 10,000 permutations of the Residual Randomisation Permutation Procedure (RRPP [43]) to generate empirical sampling distributions for significance testing, and from which effect sizes were estimated as standard deviates (z-scores).

We applied the same modelling approach to our *P*1_*phy*_ and *P*7_*phy*_ datasets but using Phylogenetic Generalised Least Squares (PGLS) rather than Ordinary Least Squares regression (OLS). Data collated from related species violate the assumption of OLS that residual error is independent between observations [44]. It is possible to account for this autocorrelation by weighting the error structure of the regression assuming a model of trait evolution to calculate covariance of traits among species [45, 46]. The default method for PGLS in ‘geomorph’ (function procD.pgls) assumes traits evolve under Brownian motion, which may not be realistic for all datasets. Therefore, we estimated Pagel’s lambda as a measure of phylogenetic signal in the residuals of our ‘full’ models using Restricted Maximum Likelihood (REML) implemented in the function ‘gls’. Using these estimated values of lambda (*P*1_*phy*_ = 0.18 and *P*7_*phy*_ = 0.13) we then calculated phylogenetic variance-covariance correction matrices and conducted PGLS with ‘procD.lm’. Calculating separate variance-covariance matrices in this way allowed us to complete all regression analyses with ‘geomorph’ functions and the RRPP method, while using a better fitting model. To infer patterns of morphospace occupation across the evolution of parasitic modes we projected our phylogeny into morphospaces to create phylomorphospaces for *P*1_*phy*_ and *P*7_*phy*_.

## Results

### Dactylus shape variation

**Fig. 2:**
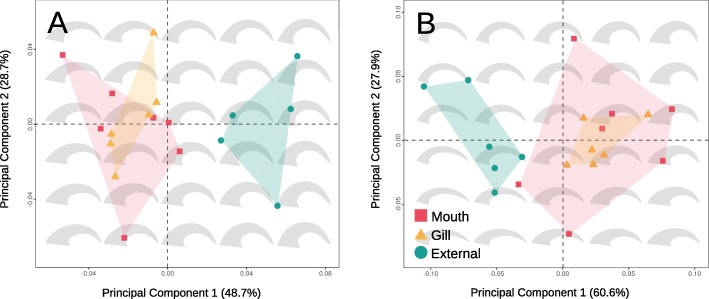
Morphospace plot of *P*1 (**a**) and *P*7 (**b**) dactyli. The first principal component is plotted on the x-axis and the second on the y-axis. Percentages included in the axis labels are the variation accounted for by each principal component. Convex hulls are calculated for each parasitic mode. Silhouettes are PCA backtransformations that depict shape across morphospaces

Principal Component Analysis (PCA) clearly separates dactylus shape of externally-attached cymothoids from gill- and mouth-attaching species for both *P*1 and *P*7, where they occupy a distinct region of morphospace (Fig. 2). The vast majority of shape variation is accounted for by the first two principal components: 77.3% and 79.3% for *P*1 and *P*1_*phy*_, 88.5% and 89.7% for *P*7 and *P*7_*phy*_. For *P*1, higher PC1 values indicate increased curvature, while PC2 reflects changes in the width of the dactylus, especially the proximal width. Interestingly, all externally-attaching species have positive PC1 values for *P*1, and gill-attaching species share similar curvatures with a narrow PC1 range. *P*7 dactyli are thinner with margins subparallel at lower values of PC1, and are flatter for negative values of PC2 (Fig. 2). Similar to *P*1, gill-attaching species occupy a small area of *P*7 morphospace, while mouth-attaching species cover the widest area, indicating a broad range of dactylus shapes.

### Relationship between dactylus shape, size and parasitic mode

Dactylus shape is significantly and strongly correlated with parasitic mode, as evidenced by the positioning of species in morphospace and from the results of Procrustes npMANCOVA (Table 2). The interaction term between size and mode has a weak and non-significant effect for *P*1 (z-score = 1.09, *p*-value = 0.86), whereas both size and mode exhibit large and significant effects (z-score = 1.92 and 2.98, respectively). For *P*7 we find no significant effect of size on shape (z-score = 0.81, *p*-value = 0.23), but mode (z-score = 2.82, *p*-value <0.001) and the interaction of size and mode (z-score = 1.66, *p*-value = 0.03) are both significant. Despite the significant interaction term in *P*7 it accounts for little of the total variance in the model (R2 = 0.09). Pairwise comparisons of angles between group allometric slopes show that the size-mode interaction in *P*7 is driven entirely by differences between external- and mouth-attaching species (z-score = 1.85, *p*-value = 0.01). As expected from results of the full model, *P*1 comparisons of group slopes show no pairwise differences. For both appendages there are significant differences between the mean shapes of externally-attaching cymothoids and the other two modes, but not between gill- and mouth-attaching species.

**Table 2:** Results from OLS regressions of the full *P*1 and *P*7 datasets

	P1			P7		
Model terms	df	z-score	*p*-value	df	z-score	*p*-value
Shape ∼ Size	1	1.92	<0.01*	1	0.81	0.23
Shape ∼ Mode	2	2.98	<0.01*	2	2.82	<0.01*
Shape ∼ Size * Mode	2	1.09	0.86	2	1.66	0.03*
	Pairwise (Group slopes)					
	E	M	G	E	M	G
E	–	0.62	0.35	–	0.01*	0.59
M	0.54	–	0.63	1.85	–	0.28
G	0.27	0.53	–	0.34	0.75	–
	z-score = 1.07, Res.df = 12			z-score = 1.53, Res.df = 13		
	*p* = 0.86			*p* = 0.05*		
	Pairwise (Group Means)					
	E	M	G	E	M	G
E	–	<0.01*	<0.01*	–	<0.01*	0.01*
M	3.78	–	0.37	3.94	–	0.94
G	3.31	0.31	–	2.62	1.33	–
	z-score = 3.08, Res.df = 124			z-score = 2.69, Res.df = 15		
	*p* < 0.01			*p* < 0.01*		
	Pairwise (Allometry-free group means)					
	E	M	G	E	M	G
E	–	<0.01*	<0.01*	–	<0.01*	<0.01*
M	3.79	–	0.37	3.82	–	0.76
G	3.32	0.31	–	3.41	0.81	–
	z-score = 3.10, Res.df = 14			z-score = 2.82, Res.df = 16		
	*p* < 0.01			*p* < 0.01*		

Group Slopes are pairwise comparisons between the allometric vectors of parasitic mode, Group Means are pairwise comparisons of Procrustes distance between modes after accounting for variation in size, and Allometry-free Group Means are the same comparisons as Group Means but using the residuals from regression of shape on size as shape variables. For pairwise comparisons, above the diagonal are *p*-values, and below the diagonal are z-scores. Below each pairwise comparison table are the values for the model (Type I Sum of Squares). E = External, G = Gill, M = Mouth

### Phylogenetic context

**Fig. 3:**
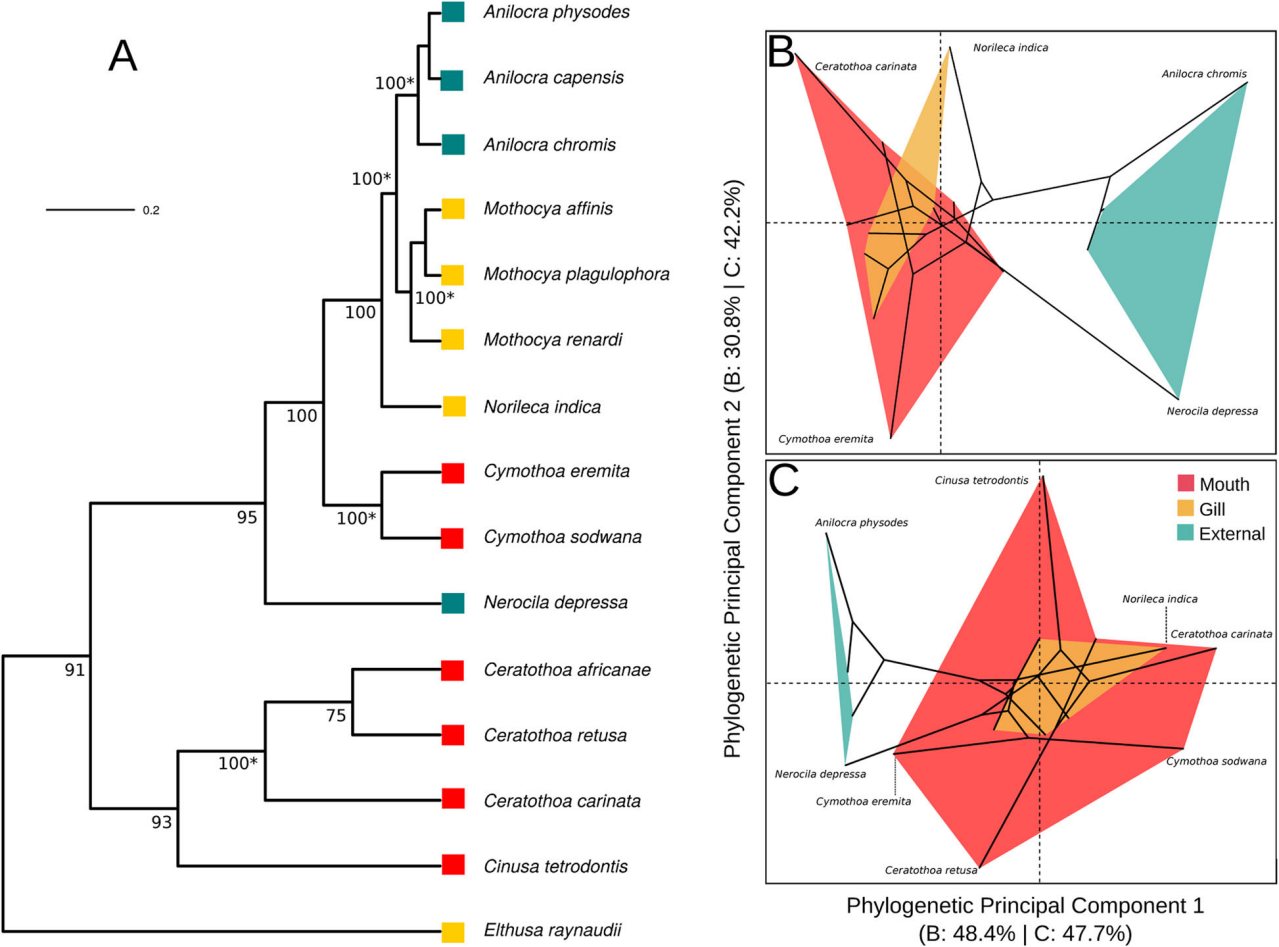
Reconstructed phylogeny (**a**), and phylomorphospace plots of *P*1_*phy*_ (**b**) and *P*7_*phy*_ (**c**) dactyli. Numbers at nodes are bootstrap support values, and asterisks are constrained nodes. For phylomorphospaces the first Principal Component is plotted on the x-axis and the second on the y-axis. The percentages included in the axis labels are the variation accounted for by each Principal Component. Convex hulls are plotted for each parasitic mode

We did not find any significant differences between the log-likelihoods of our constrained and unconstrained topologies using Shimodaira-Hasegawa test(diff = -0.03, *p* = 1) (Fig. 3a). As expected, the constrained topology is consistent with that of [12] except we do not recover a sister relationship between *Cymothoa* and *Nerocila*. Evidence from previous work continues to show that evolution of attachment mode in cymothoids is homoplastic, with gill and external attachment likely to have arisen independently at least twice or for there to have been secondary reversals [12, 13]. The first two components of pPCA for *P*1_*phy*_ account for 48.4% and 30.8% of variation in shape respectively, while for *P*7_*phy*_ pPC1 reflects 47.7% and pPC2 42.2%. Mouth-attaching genera occupy a much greater portion of phylomorphospace than other modes and exhibit similarly striking patterns as to our full datasets: *Cymothoa eremita*, for example, exhibits a *P*7 morphology most similar to external species than to the congener *C. sodwana*, (Fig. 3c). Gill-attaching species have highly similar *P*7 morphologies but *Norileca indica* has a *P*1 morphology distinct from the other gill-attaching species, perhaps due to its unusual attachment orientation on the ventral side of the operculum, facing anteriorly [47]. Most gill-attaching species are found anchored to the gill filaments with their dorsal side facing the operculum. Accounting for phylogeny with PGLS results in large and significant shape differences between parasitic modes for both *P*1_*phy*_ and *P*7_*phy*_ (Table 3). There is no evidence of separate allometric slopes between groups for either dataset using PGLS but, as with *P*1, allometry remains an important influence on shape for *P*1_*phy*_. We find the same pattern in pairwise comparisons as with OLS regression from our full datasets, where the mean shapes of gill- and mouth-attaching species are significantly different from externally-attaching species, but not from each other.

**Table 3:** Results from PGLS regressions of the *P*1_*phy*_ and *P*7_*phy*_ datasets

	P1			P7		
Model terms	df	z-score	*p*-value	df	z-score	*p*-value
Shape ∼Size	1	1.96	0.01*	1	0.42	0.36
Shape ∼Mode	2	2.02	0.01*	2	1.70	0.04*
Shape ∼Size * Mode	2	1.66	0.96	2	1.25	0.10
	Pairwise (Group slopes)					
	E	M	G	E	M	G
E	–	0.82	0.44	–	0.01*	0.57
M	0.96	–	0.43	1.78	–	0.24
G	0.10	0.01*	–	0.27	0.91	–
	z-score = 1.71, res.df = 9			z-score = 1.22, res.df = 9		
	*p* = 0.96			*p* = 0.11		
	Pairwise (Group means)					
	E	M	G	E	M	G
E	–	0.01*	0.02*	–	0.01*	0.04*
M	2.64	–	0.36	2.77	–	0.97
G	2.29	0.31	–	1.89	1.49	–
	z-score = 2.31, res.df = 11			z-score = 1.61, res.df = 15		
	*p* = 0.01*			*p* = 0.04*		

Group Slopes tables are pairwise comparisons between the allometric vectors of parasitic mode and Group Means tables are pairwise comparisons of Procrustes distance between modes after accounting for variation in size. For pairwise comparisons, above the diagonal are *p*-values, and below the diagonal are z-scores. Below each pairwise comparison table are the values for the model (Type I Sum of Squares). E = External, G = Gill, M = Mouth

## Discussion

Our results indicate that the shape of cymothoid dactyli are strongly influenced by parasitic mode corroborating theory that adaptation to hosts and local environments is a driver of parasite phenotypes [48]. Other empirical studies also link the morphology of parasite attachment to various ecological factors: in relation to host size in feather lice [49]; host specialisation in platyhelminth fish parasites [50]; host thermal regulation strategy in acanthocephalans [51]; host biogeographic plasticity [52]; and, as we also find here, microhabitat [53, 54]. However, phylogeny may also explain morphological variation where more closely related species are expected to share similar traits. For example, [55] and [56] both suggest that integration between parts of monogenean attachment organs results in phylogenetic constraint of shape variation. Both of these studies find that divergent parasite species that infest the same host species possess differently shaped attachment organs (called haptors). We have found no evidence that there are clade-specific patterns of association between parasite mode and dactylus shape. Our results imply that different clades can converge on the same dactylus shape, which presumably is well-adapted for that particular parasite mode.

Large effect sizes are consistently observed for parasitic mode in both OLS and PGLS analyses of *P*1 and *P*7 datasets. Underlying this pattern are significant shape differences between externally-attaching species, and both gill- and mouth-attachers. Interestingly, however, the shape dissimilarities of external and internal species in morphospace are also different between *P*1 and *P*7 dactyli. We also find that the shape of *P*1 dactyli is influenced by size, but this is not the case for *P*7. Altogether, this suggests that anterior and posterior dactyli may function differently, and in externally-attaching species the differences are particularly acute. Certainly, pereopods are broadly arranged in two opposing angles between pereopods 1-3 and 4-7, which is suggested to enhance attachment ability [8, 14]. *P*1 dactylus shapes are highly recurved, whereas for *P*7 the shape is flatter and more slender than those of gill- and mouth-attaching species. Gill- and mouth-attaching species, on the other hand, possess more similar *P*1 and *P*7 morphologies. All isopods exhibit biphasic moulting [57]; a particularly useful trait for cymothoids in that they do not need to completely detach from the host during ecdysis. It has previously been suggested that this preadaptation may have been key to isopods evolving ectoparasitic lifestyles [14]. The risk of detachment during ecdysis is presumably far greater for external-attaching species and posterior dactyli could provide secure anchorage if they function to pierce host tissue, as *P*7 morphology suggests they might.

Inhabiting the external environment seems to result in convergence of *P*7 dactylus morphology, where relatively less related species share similar morphologies. External-attaching species have a narrow range of PC1 scores, and *Anilocra capensis* shares a more similar morphology with *A. chromis* and *Nerocila depressa* than its closest relative in our phylogeny, *A. physodes*. Similarly, gill-attaching species share very similar *P*7 morphologies. Welicky et al. [58] and Pawluk et al. [59] have shown that cymothoid and host size are tightly correlated except for gill-attaching species. This suggests that available space in the opercular cavity does not scale linearly with host size much in the same way that, to draw parallels with eye anatomy, the upper and lower tarsi are near flush to the sclera or cornea regardless of size. Such a restriction could limit growth patterns of gill-attaching species, including their dactyli. Morphology of gill-attaching species appears to neatly fit their location and orientation within the gill cavity, where they are typically asymmetrical, twisted to one side, and either completely flat or extremely concave. Therefore, there could be two constraints operating on *P*7 morphology: exposure and detachment risk related to external parasitism, and for gill-attaching species the volume of the opercular cavity.

We observe the opposite pattern in mouth-attaching species, which exhibit highly divergent *P*7 forms. Free from hydrodynamic forces and the risks of being brushed or cleaned off, a fish mouth is, perhaps counter-intuitively, a safe environment in which to reside as an ectoparasite. Mouth-attaching species have adapted to ’bite tongues’ but they also secure themselves to the upper palate, as in *Cinusa tetrodontis* [18], wrap their dactyli around the tongue, or attach to the inner cheek and gill arches. Most mouth-attaching species are oriented anteriorly, but a few species face posteriorly into the throat, like all species in the genus *Isonebula* [60]. Therefore, mouth-attachment as a discrete parasitic mode actually conceals considerable microhabitat variation that could explain the diversity of *P*7 dactylus shapes. In addition, host specificity and the manner of parasite speciation might also be important. Except for *Cymothoa eremita*, each mouth-attaching species in our dataset is known from fewer than three host species, and even *C. eremita* shows strong preference toward *Parastromateus niger* [5, 20]. Host switching is a common model explaining parasite speciation, but only a subset of potential new hosts may be a suitable match for parasite phenotypes and it is likely that new host environments are initially suboptimal. After a host-switch event, subsequent co-adaptation between the parasite and the new (suboptimal) host environment would then refine morphological features, potentially driving shape differences between parasite species.

Another possible determinant of cymothoid attachment morphology is feeding ecology. It has previously been suggested that while externally-attaching species depredate hosts, feeding on blood and tissue, gill- and mouth-attaching species may additionally take up energy from prey their hosts consume [61–64]. Certainly, the relatively benign effects of many gill- and mouth-attaching species could support this view [65, 66]. Differences in feeding ecology are also consistent with mouthpart morphology, where gill- and mouth-attaching cymothoids possess molar processes and incisors adapted to grind or slice tissues. Externally-attaching species, however, have mouthparts that interlink to form a functional sucking cone, adapted for fluid intake [14]. In externally-attaching species, the incisor process is narrowed to a point rather than a blade, perhaps for piercing host flesh, while the mouthparts are angled downward toward the host. Furthermore, the primary mouthparts are restricted in their movement, only able to motion in an inward-outward direction. Such a movement requires a suitable counter-force to hold the mouth close to the host [14]; a role that might be performed by the anterior pereopods and, in part, determine the *P*1 shape of externally-attaching species.

Size is also a significant predictor of *P*1 shape in OLS and PGLS analyses. As stated previously, the external environment must impose unique challenges, not least of which is hydrodynamic force. We suggest that the directionality of this force could be key to understanding the different influences of size between *P*1 and *P*7 dactylus shapes. All externally-attaching species orientate anteriorly, parallel with their hosts [10, 16]. In our dataset, they also possess the largest and most recurved *P*1 dactyli (mean centroid size = 7.54). The anterior dactyli in cymothoids are angled in a manner ideal for pulling against the direction of flow, which requires less effort than pushing due to reduction of frictional force. Such efficiency is likely complemented in externally-attaching species by increased strength derived from *P*1 dactylus shape and size. Drawing parallels from structural engineering, we can conceive that steeper arches with more symmetrical parabolic geometries are able to resist greater forces in compression [67]. Gill- and mouth-attaching species, on the other, hand are not subject to the same demands of the external environment, and possess *P*1 dactyli that are not as recurved and are perhaps better suited to withstand other structural displacements like shear.

## Conclusions

We used geometric morphometrics, multivariate analyses, and phylogenetic comparative methods to quantify shape variation in the attachment structures of cymothoid isopods to determine whether shape differences between species correspond to parasite mode. In addition, we assessed the relative influences of allometry and shared ancestry. We found that ecology is the primary driver of dactylus shape. Separate lineages appear to have independently evolved similar dactylus morphologies that are presumably optimal for particular parasitic modes. The clearest differences are between externally-attaching species, and those attached internally in the gill or mouth. Geometric morphometrics is a powerful method for uncovering complex patterns from simple outline shapes like dactyli. Of particular note are the shape differences we found between anterior and posterior pereopod shapes. These anterior-posterior differences are also characteristic of the different parasitic modes and likely reflect adaptations to obligate parasitism and differences in feeding ecology between externally-attaching, and gill- and mouth-attaching cymothoids.

## Data Availability

The datasets generated during the current study and scripts for their analysis are available in the following github repository: https://github.com/bailliecharles/hooks. Files include raw landmark files, our phylogenetic tree in Newick format and R scripts for completing analyses. Sequences can be retrieved from Genbank with accession numbers provided in Table 1. All files will also be made available promptly upon request by contacting the authors directly.

## Abbreviations

AICc: Akaike’
s information criterion corrected; ANOVA: Analysis of variance
COI: Cytochrome oxidase subunit I
DNA: Deoxyribonucleic acid
GTR: General time reversible
MSE: Mean squared error
npMANCOVA: Non-parametric multivariate analysis of covariance
PC: Principal component
PCA: Principal component analysis
PCR: Polymerase chain reaction
PGLS: Phylogenetic generalised least squares
pPC: phylogenetic principal component
OLS: Ordinary least squares
